# Effect of film thickness in gelatin hybrid gels for artificial olfaction

**DOI:** 10.1016/j.mtbio.2019.100002

**Published:** 2019-03-22

**Authors:** Carina Esteves, Gonçalo M.C. Santos, Cláudia Alves, Susana I.C.J. Palma, Ana R. Porteira, João Filho, Henrique M.A. Costa, Vitor D. Alves, Bruno M. Morais Faustino, Isabel Ferreira, Hugo Gamboa, Ana C.A. Roque

**Affiliations:** a UCIBIO, Departamento de Química, Faculdade de Ciências e Tecnologia, Universidade Nova de Lisboa, Caparica, Portugal; b LIBPhys-UNL, Departamento de Física, Faculdade de Ciências e Tecnologia, Universidade Nova de Lisboa, Caparica, Portugal; c LEAF – Linking Landscape, Environment, Agriculture and Food, Instituto Superior de Agronomia, Universidade de Lisboa, Lisboa, Portugal; d CENIMAT/I3N, Departamento de Ciências dos Materiais, Faculdade de Ciências e Tecnologia, Universidade Nova de Lisboa, Caparica, Portugal

**Keywords:** Gelatin, Ionic liquid, Liquid crystal, Gas sensor, Electronic nose, Machine learning

## Abstract

Artificial olfaction is a fast-growing field aiming to mimic natural olfactory systems. Olfactory systems rely on a first step of molecular recognition in which volatile organic compounds (VOCs) bind to an array of specialized olfactory proteins. This results in electrical signals transduced to the brain where pattern recognition is performed. An efficient approach in artificial olfaction combines gas-sensitive materials with dedicated signal processing and classification tools. In this work, films of gelatin hybrid gels with a single composition that change their optical properties upon binding to VOCs were studied as gas-sensing materials in a custom-built electronic nose. The effect of films thickness was studied by acquiring signals from gelatin hybrid gel films with thicknesses between 15 and 90 μm when exposed to 11 distinct VOCs. Several features were extracted from the signals obtained and then used to implement a dedicated automatic classifier based on support vector machines for data processing. As an optical signature could be associated to each VOC, the developed algorithms classified 11 distinct VOCs with high accuracy and precision (higher than 98%), in particular when using optical signals from a single film composition with 30 μm thickness. This shows an unprecedented example of soft matter in artificial olfaction, in which a single gelatin hybrid gel, and not an array of sensing materials, can provide enough information to accurately classify VOCs with small structural and functional differences.

## 1 Introduction

Gas sensing is an emerging field in medicine, particularly for the development of non-invasive diagnostic tools [1], [2], [3], [4]. Electronic noses (e-noses) are user-friendly and portable gas-sensing devices, comprising an array of sensing probes, which generate measurable signals in the presence of volatile organic compounds (VOCs). The signals are further processed and analyzed using pattern recognition tools, which ultimately allow the identification of characteristic VOC profiles associated to a particular sample. As such, e-noses conceptually mimic the sense of olfaction, in a perfect combination between chemical sensing and artificial intelligence, mirroring the biological orchestra of olfactory proteins and the intricate brain computing processes used in odor recognition [5]. The most conventional e-nose sensing materials are metal oxide semiconductors and synthetic conducting polymers. These are associated with low selectivity, sensor drift, and high operating temperatures [6], which has been triggering the search for alternative gas sensors, either through the incorporation of biological components from natural olfaction or through the use of stimuli-responsive biomaterials, including gelatin [7].

Arrays of gelatin ionogels with varied ionic liquids (ILs) have been previously described as electrical gas-sensing probes. These arrays were tested with 7 different VOCs, and through processing of the peak relative amplitude with principal component analysis (PCA), a good clustering of the different VOCs was observed [8]. Recently, our group introduced the concept of hybrid gels, consisting of biopolymeric matrices encapsulating IL–liquid crystal (LC) self-assembled droplets. Hybrid gels combine the ionic conductivity of ILs with the unique optical properties of LCs, resulting in optoelectrical stimuli-responsive materials. An array of three hybrid gels, varying in the biopolymer (gelatin and dextran) and IL (1-butyl-3-methylimidazolium dicyanamide [BMIM][DCA] and 1-allyl-3-octylimidazolium chloride [ALOCIM][Cl]), was tested with 11 distinct VOCs. Using the peak relative amplitude from the signals, PCA showed an efficient clustering of the different VOCs [9].

In this work, the potential of gelatin as the material of choice to generate hybrid gels for optical gas sensing is further evaluated. By simultaneously optimizing the hybrid gels film thickness and implementing a dedicated automatic classifier based on support vector machines (SVM) for data processing, it is shown that a single gelatin hybrid gel composition with defined thickness can classify with high accuracy a set of 11 VOCs with small structural and functional differences. This is the first report of a gelatin-based hybrid material with application in intelligent gas sensing. Our findings further strengthen the position of soft biomaterials as the basis to design advanced functional materials with applications in artificial olfaction, biomedicine [10], and soft optoelectronics [11].

## 2 Materials and methods

### 2.1 Materials and reagents

Gelatin from bovine skin (gel strength ≈225 g; Bloom, Type B), glutaraldehyde solution (50 wt % in water), fluorescein isothiocyanate isomer I, and sodium bicarbonate were purchased from Sigma-Aldrich. Dimethyl sulfoxide was obtained from Fisher Scientific. The LC 4-cyano-4′-pentylbiphenyl (5CB) was acquired from TCI Europe, and the IL [BMIM][DCA] (˃98%) was purchased from IoLiTec. The solvents dichloromethane and hexane were purchased from VWR, and ethanol (purity ≥99.8%) was purchased from Sigma-Aldrich. Acetonitrile (purity ≥99.9%), chloroform, diethyl ether (HPLC grade), ethyl acetate, heptane, methanol (HPLC grade), and toluene were supplied by Fisher Scientific. Acetone (purity ≥99.5%) was purchased from Honeywell, and isopropanol (purity ≥99.5%) from ROTH. Solvents were of analytical grade and used as received.

### 2.2 Production of hybrid gel films

The protocol described in the studies by Hussain et al. [9] and Semeano et al. [12] was followed. Briefly, the IL ([BMIM][DCA]) and LC (5CB) were mixed by magnetic stirring, after which gelatin and milliQ water were added. The mixture was then deposited onto an untreated glass slide, using an automatic film applicator equipped with a heated bed and a quadruplex with four different predefined thicknesses: 15 μm, 30 μm, 60 μm, and 90 μm (TQC Sheen). The hybrid gel films were then left at room conditions for 24 h before characterization, unless otherwise stated. For the preparation of glutaraldehyde cross-linked hybrid gels, the same protocol was followed except that a glutaraldehyde solution (5 wt % in water) was added to the mixture after the addition of milliQ water.

### 2.3 Characterization of hybrid gel films

The thickness of hybrid gel films was determined by optical microscopy one week after production. A small incision was made in each gel film to remove a corner, as illustrated in Fig. S1a. The samples were observed by optical microscopy (Zeiss Axio Observer.Z1/7 coupled with an Axiocam 503 color camera), and the thickness of each thin film was determined using the ZEN software, version 3.2, measurement tool. For thickness determination, a three-dimensionally printed adapter was made to allow the placement of the glass slide in an orientation of 75° regarding the microscope stage (Fig. S1b). With this configuration, the surface of the gel film can be scanned using the Z-axis, avoiding the interference of other focal planes as it would happen if the placement was made in an orientation of 90°. To compensate for this deviation to the parallel plane of the incision, Eq. (1) is used to determine the measured thickness.

(1) $$Measured\;thickness=\frac{Acquired\;thickness\left(ZEN\right)}{\text{Cos}\left(90-75\right)}$$

To observe the optical textures of the LC droplets and the morphology of the gel films, polarized optical microscopy (POM) images were taken under crossed (at 90°) and semicrossed (at 45°) polarizers in a Zeiss Axio Observer.Z1/7 microscope equipped with an Axiocam 503 color camera and ZEN 2.3 software for image acquisition and processing.

**Fig. 1:**
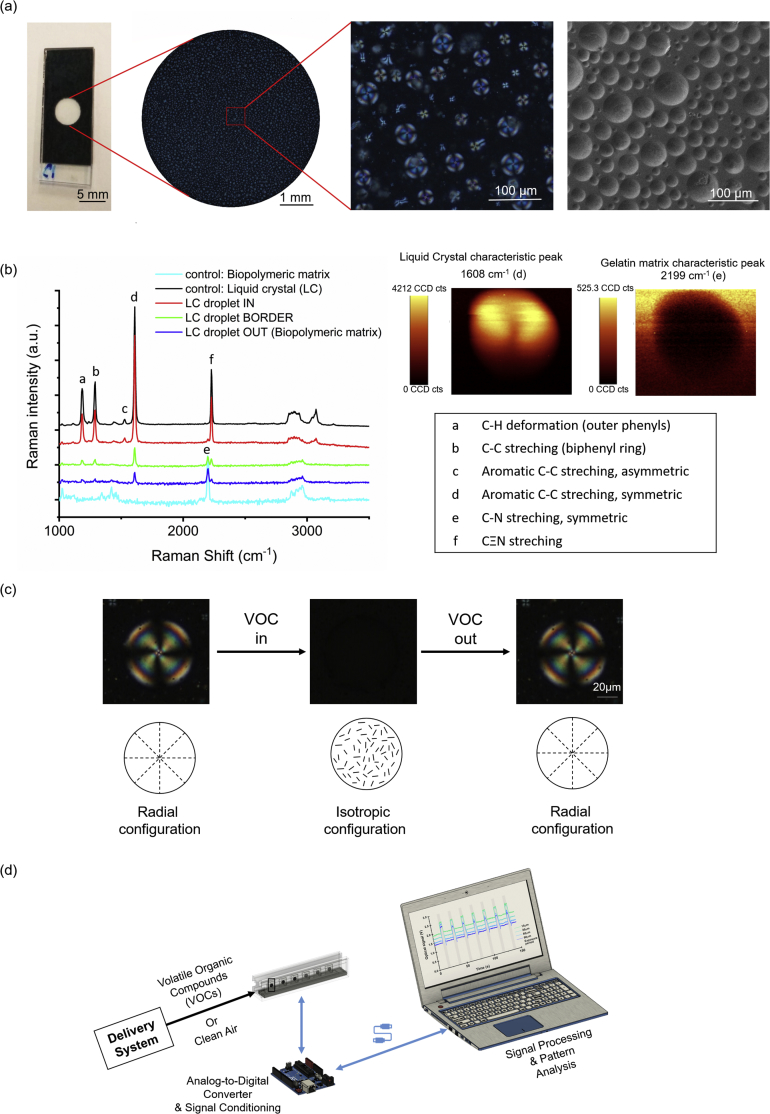
Morphological and optical properties of hybrid gels and schematics of in-house–developed electronic nose. a) Image of hybrid gel films in real scale, the definition of the optically active area, and examples of typical images obtained by polarized optical microscopy (POM) and scanning electron microscopy. b) Detailed composition analysis of a liquid crystal (LC) droplet encapsulated within the hybrid gel film, obtained by Raman spectroscopy. Relevance is given to the distribution of the characteristic liquid crystal and matrix peaks in a droplet [31], [32]. c) Analysis of a single LC droplet observed by POM before, during, and after exposure to air saturated in hexane. d) Schematic representation of the in-house–built electronic nose.

To determine the optically active area of each film, the back side of glass substrates was covered with a black mask with a circular hole with a diameter of 5 mm [13], as shown in Fig. 1a. The optically active area of a film was defined as the birefringent area that contributes to the optical signal generated by the sensing area of the film. To quantify the optically active area, a crossed polarizers panoramic POM image of the sensing area (Fig. 1a) was first generated using the “Tiles” module of the ZEN software. Then, the mean gray value of the circular area in the panoramic image was measured using the tools of FIJI distribution [14] of ImageJ open software [15].

To observe the compartmentalization of LC droplets inside the gelatin-IL matrix, the surface of hybrid gel films was analyzed by Raman spectroscopy using a Witec Alpha 300 confocal RAS with a 532-nm argon laser at 0.5 mW of power.

### 2.4 Observation of hybrid gel films during VOC exposure by POM

Hybrid gel films with 30 μm thickness were placed in an in-house–designed glass chamber, fixed under a polarized optical microscope (Zeiss Axio Observer.Z1/7 equipped with an Axiocam 503 color camera) and sequentially exposed to the headspace of 7 organic solvents (hexane, toluene, dichloromethane, diethyl ether, acetone, ethanol, and isopropanol) previously heated to 37 °C in a thermostatized water bath for 15 min. For each exposure/recovery cycle, videos of 40 s were recorded with 90° and 45° crossed polarizers. The gels were initially stabilized through exposure to room air for 10 s. Afterward, 10 ml of the solvent's headspace (35 ml for ethanol and isopropanol) was pushed with a syringe and inserted into the glass chamber containing the hybrid gel film. Fifteen seconds later, an air pump was used to regenerate the system with ambient air. The optical sensor response was determined by the variation of the intensity of transmitted light through the gel along time, calculated using ImageJ software for the determination of the mean gray value.

### 2.5 Acquisition of optical signals from films upon exposure to VOCs

Hybrid gel films were used as sensing materials in an in-house tailor-made e-nose. The e-nose device consists of a sensors chamber, isolated from ambient air and light, including six independent sensor slots, each of them composed by a pair of aligned light emitting diode and a photodiode, between which the sensing gel is placed sandwiched by two polarizers crossed at 90° (Fig. 1d). Two pumps work alternately. The exposure pump pushes the VOC into the chamber, and the recovery pump injects ambient air to clean the chamber. When VOCs interact with the gel, it loses its ability to rotate polarized light and therefore, the intensity of light that hits the photodiode changes. The change in the light intensity read by the photodetector during exposure/recovery cycles is digitalized using an Arduino Due, and stored for subsequent analysis.

Four out of the six independent sensor slots of the e-nose sensor chamber were occupied with gelatin hybrid gel films with distinct thicknesses (15 μm, 30 μm, 60 μm, and 90 μm), a thickness per slot. Then, the films were exposed sequentially to the headspace of 11 solvents (heptane, hexane, chloroform, toluene, dichloromethane, diethyl ether, ethyl acetate, acetonitrile, acetone, ethanol, and methanol) previously heated to 37 °C in a thermostatized water bath for 15 min. The concentration of each VOC in the sensor chamber was calculated between 12 and 15% (v/v) as explained in Supplementary Info. The films were exposed to each VOC for 45 consecutive cycles (each cycle: 5 s exposure to VOC, 15 s recovery with air) in contiuous assays with duration of 15 min. Optical signals of the four sensing films were acquired at a sampling rate of 90 Hz. Assays were performed in duplicate. The as-generated data for each film thickness was then independently analyzed using computing processes and machine learning tools (Section 2.6).

### 2.6 Signal processing and automatic VOC classification

Signals from each film thickness were first filtered using a smooth filter (with a sliding window of 100 points) from novainstrumentation Python library (https://github.com/hgamboa/novainstrumentation) and then divided into cycles; this allowed to treat each cycle independently regarding the same film thickness. Twelve features were extracted per cycle (Fig. S2) and used as input variables for an automatic classifier algorithm based on SVM. While the first 9 features were derived from morphology of the cycle signal and respective derivatives [16], features 10 to 12 were derived from fitting the cycle signal to the logistic function. In total, there were 90 cycles per VOC for each gel film thickness; this data set was split in training and testing sets for the SVM classifier to learn a VOC classification model The SVM was tuned with the radial basis kernel and hyperparameters C = 100 and γ = 0.1. Stratified 10-fold cross validation was used, and the classification results for each thickness (15 μm, 30 μm, 60 μm, and 90 μm) were represented in normalized confusion matrices.

## 3 Results and discussion

### 3.1 Hybrid gelatin films with distinct thickness

Gelatin is a biopolymer derived from the hydrolysis of collagen, a protein typically extracted from the bones and skin of bovine, pig, or fish. Gelatin molecules undergo conformational changes from coil to triple-helix structure during the thermal gelation process, resulting in hydrogels [17]. It is known that gelatin can also form ionogels when dissolved in ILs, yielding robust and versatile materials. The hybrid gelatin gels described in this work add an extra component to ionogels as they consist of gelatin, the IL [BMIM][DCA], and the LC 5CB in the presence of water (∼10% [w/w]) [18]. This unique composition generates physical compartmentalization within the gels because the LC molecules remain encapsulated in droplets with a typical radial configuration, triggered by the presence of the IL, which simultaneously dissolves gelatin (Fig. 1a and b and Fig. S5). Such materials possess ionic conductance because of the presence of IL molecules and also interesting optical properties conferred by the presence of LC. LC molecules are a class of soft matter because they possess molecular order and can be aligned along an axis. Aligned LCs are able to manipulate the polarization of light, allowing the transmission of light in optical devices and the interference of colors seen under POM (Fig. 1a). Furthermore, such molecular order is easily perturbed by physical and chemical stimuli, and as such, LCs can act as amplification optical probes. When exposed to VOCs, a disruption in the orientation of LC molecules within the droplet is observed, leading to a change from radial to isotropic configuration. The initial radial configuration is then recovered when exposed to clean air, which is clearly observed by POM (Fig. 1c). This shows the remarkable dynamic molecular rearrangement of LCs in droplets as a function of fast adsorption-desorption of VOCs into the hybrid gel films, as well as a supramolecular memory effect in hybrid gels during the reversible VOC interaction. It is important to note that the radial configuration of LCs is particularly relevant to yield a fast and dynamic optical response upon exposure to VOCs. In fact, LC droplets within gelatin hydrogels reveal a bipolar configuration, clearly less responsive to VOC stimuli when compared with LC droplets in radial configuration encapsulated in ionogels (Videos S1 and S2. Supplementary video related to this article can be found at https://doi.org/10.1016/j.mtbio.2019.100002).

The following are the supplementary data related to this article:

Video S1 Video S1

Video S2 Video S2

**Fig. 2:**
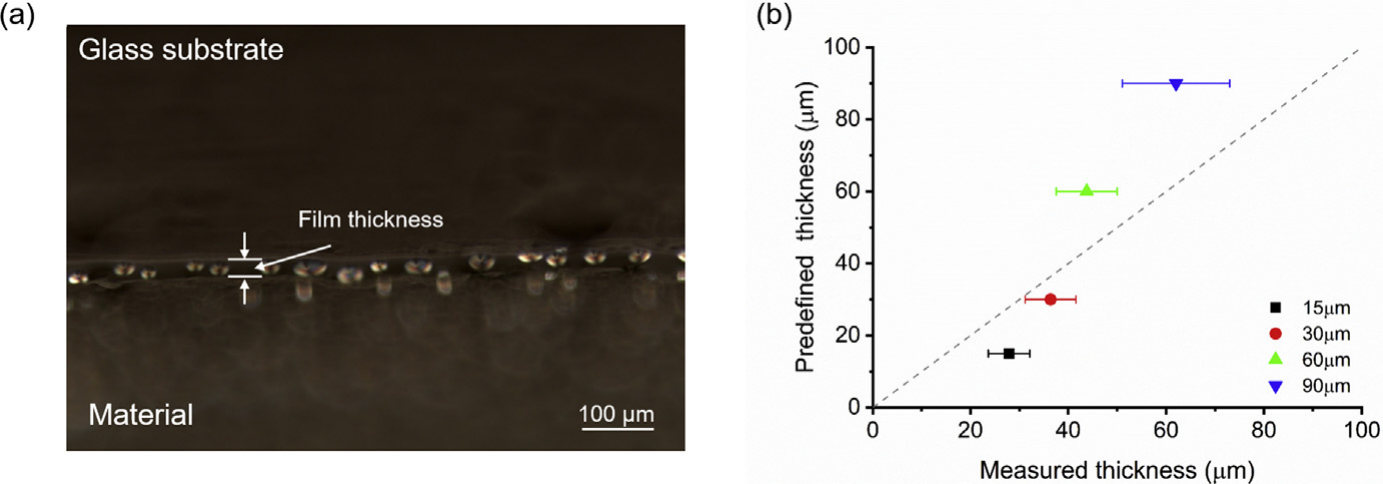
Hybrid gel film thickness. a) Cross-sectional observation by polarized optical microscopy of a hybrid gel film, where the physical encapsulation of liquid crystal droplets is visible. b) Correlation between the predefined thickness of hybrid gel films and the measured thickness (n = 3).

The adsorption-desorption processes of VOC molecules in materials are influenced by the film thickness. To evaluate this effect on gelatin hybrid gels, films with distinct predefined thicknesses (15 μm, 30 μm, 60 μm, and 90 μm) were deposited onto untreated glass substrates. Owing to the softness of the hybrid gel films when handled, although presenting mechanical spectra revealing well-structured matrices (Fig. S5), the thickness of the films was estimated by optical microscopy. Upon performing a cut in the films, and by observing the cross-section under the microscope, the limits of the film in contact with the glass substrate and with air could be delineated. Interestingly, it was also possible to observe the encapsulation of the LC droplets within the gel (Fig. 2a). When comparing the predefined film thickness with the measured film thickness, it is noticeable that 15-μm-thick and 30-μm-thick films have a measured thickness 1.2–1.8 times higher than the predefined thickness, and that the 30-μm-thick films present the smaller deviation (20%). On the other hand, 60-μm-thick and 90-μm-thick films present a smaller measured thickness, 44 μm and 62 μm, respectively (Fig. 2b). Gelatin hydrogels possess high water contents (75% [w/w]), whereas gelatin hybrid gels possess much lower water contents (10% [w/w]) because the IL also dissolves the gelatin and promotes the gelation process, replacing water. ILs have a low vapor pressure, and as such, the robustness of ionogels and hybrid gels to cracking and storage is high [9]. Still, the IL used in this work, [BMIM][DCA], as well as the gelatin itself are in equilibrium with water in the environment, resulting in slight gel swelling and shrinking.

### 3.2 Optical properties of films with distinct thicknesses

To obtain easily processed signals from the dynamic molecular rearrangements of LCs in droplets upon exposure to VOCs, the gels were used as sensing materials in a tailor-made e-nose (Fig. 1d). When the hybrid gels are deposited as films, an uncovered region is defined in the glass substrates, allowing the definition of an optically active area, which corresponds to the area where light is able to travel between the light source and the photodetector sandwiched by crossed polarizers (Fig. 1a). The LC transitions triggered by the presence of VOCs lead to different light intensities detected by the photodetectors, resulting in a variation in the measured electrical signal. In practice, the photodiode circuit implies that more light reaching the photodiode corresponds to a lower signal.

**Fig. 3:**
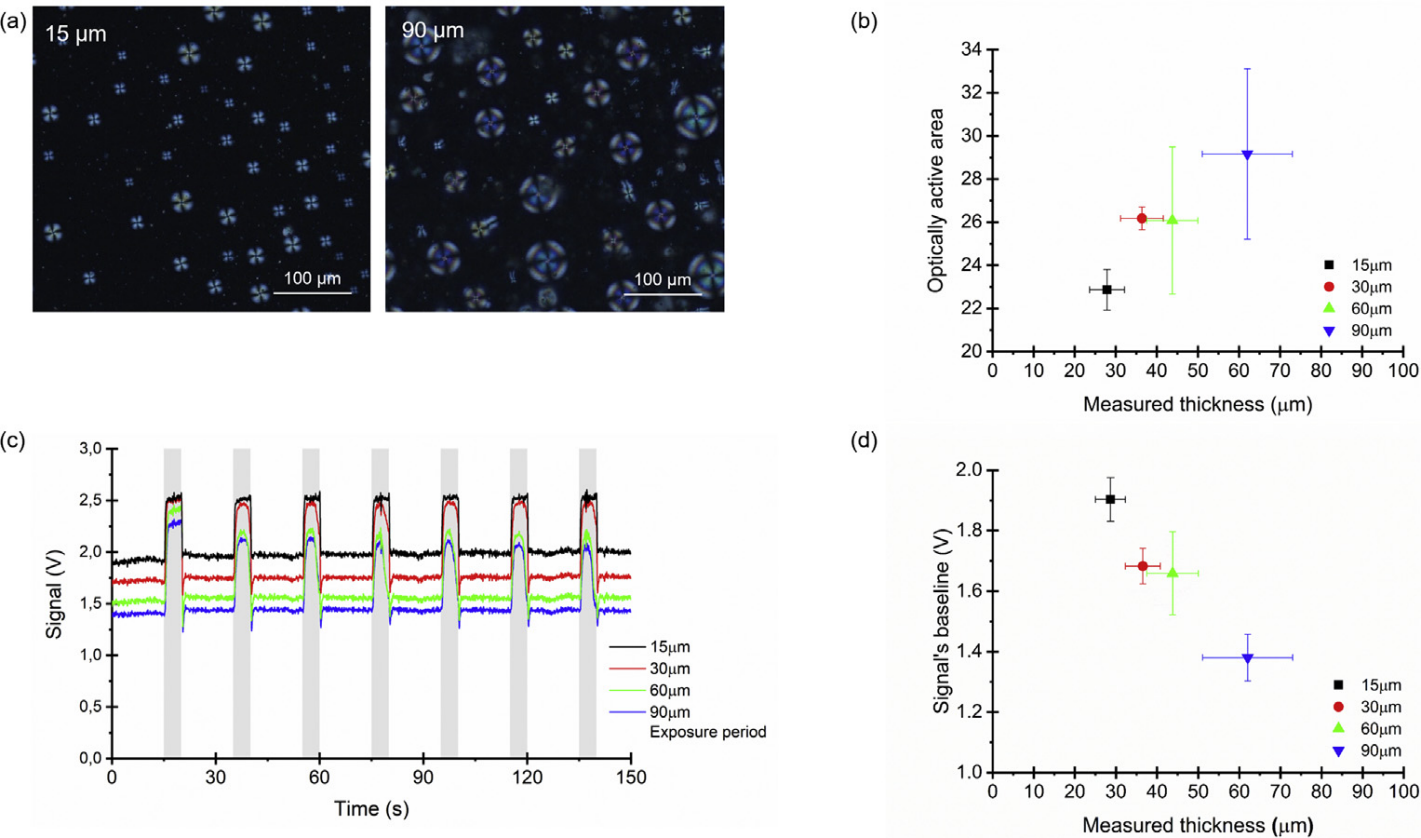
How hybrid gel film thickness relates to the optical active area for sensing. a) Examples of polarized optical microscopy images obtained for the 15-μm- and 90-μm-thick films. b) The correlation between optically active area and measured thickness of the hybrid gel films (n = 3). c) Typical signals obtained from hybrid gel films with distinct thicknesses upon cycles of heptane exposure and recovery. d) Variation of signal baseline with the measured thickness of the hybrid gel films (n = 3).

It was observed that the optically active area of films with distinct thicknesses was different (Fig. 3a and b). In general, the optically active area was lower for thinner films and higher for thicker films. This is mostly related with the diameter of the LC droplets, which tends to be higher for thicker films (Fig. S2), and also with the existence of more microscope-focusing layers as the film thickness increases. This tendency is also observed in the signals obtained from the films with distinct thicknesses. When the film thickness is higher, the optically active area is higher, and as a result, the baseline signal is lower as more light reaches the photodiode (Fig. 3c and d).

### 3.3 Optical response of hybrid gelatin films to VOCs

**Fig. 4:**
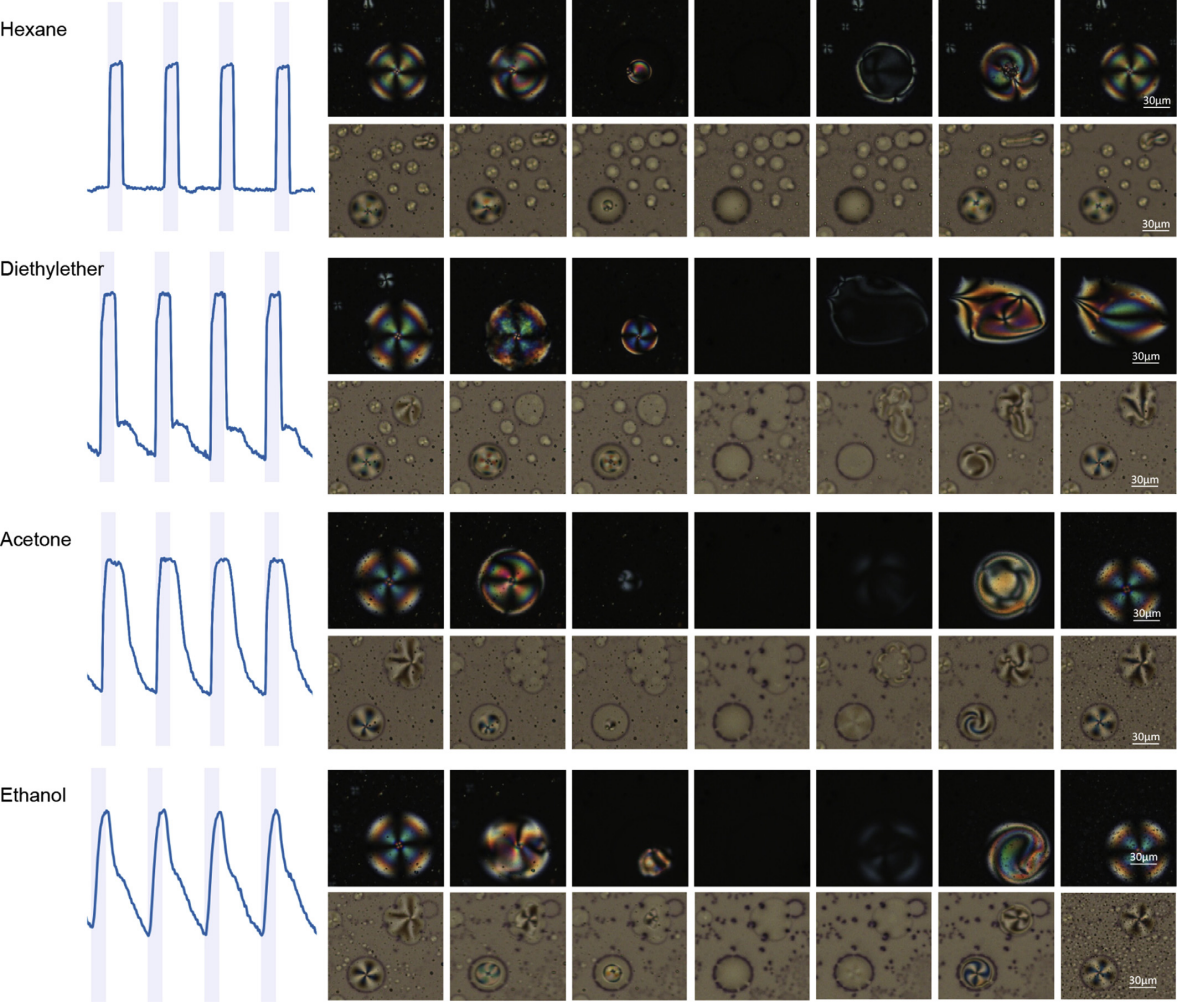
Optical signatures of the response of hybrid gelatin films with 30 μm thickness to distinct volatile organic compounds (VOCs) at 12%–15% (v/v) in the sensors chamber. Examples are given for four distinct VOCs (hexane, diethyl ether, acetone, and ethanol), showing the typical profile of the signals obtained during exposure and recovery cycles in the electronic nose (left side), as well as the corresponding optical and morphological changes observed in the hybrid gels by polarized optical microscopy with 90° crossed polarizers (top) and 45° crossed polarizers (bottom).

The hybrid gelatin films are versatile in the sense that the different compartments formed within the gel potentiate the partitioning of VOC molecules. When the gelatin films are exposed to different VOCs, the patterns of disorganization and reorganization of LC molecules during exposure and recovery, respectively, are distinct. Interesting morphological changes upon VOCs exposure can be observed by POM not only with 90° crossed polarizers but also with 45°, revealing details not related with the anisotropic LC (Fig. 4). Hydrophobic VOCs, such as hexane, are likely to interact mainly with the oil phase formed by LC molecules inside the droplets. Protic VOCs, and those forming hydrogen bonds (e.g., diethyl ether and ethanol), tend to interact not only with the LC droplets but also with the gelatin matrix itself, as previously reported [9]. LC droplets within the gelatin films are very stable over time, indicating good matrix stability (Fig. S7). However, during exposure to ethanol, a matrix reorganization is observed. This lead us to hypothesize that cross-linking the gelatin with glutaraldehyde could reinforce the matrix and increase its mechanical robustness, stability to storage, and optical response to VOCs (Figs. S5, S6–S8). However, no differences were observed between non–cross-linked and cross-linked gelatin hybrid films, which may be probably related with gelatin self-assembly in the presence of IL, that differs from conventional hydrogelation mechanisms. In fact, ILs, such as the [BMIM][DCA] used in this work, can interact with gelatin chains mainly through ionic interactions, which may impair glutaraldehyde cross-linking through free lysine residues [9], [18].

**Fig. 5:**
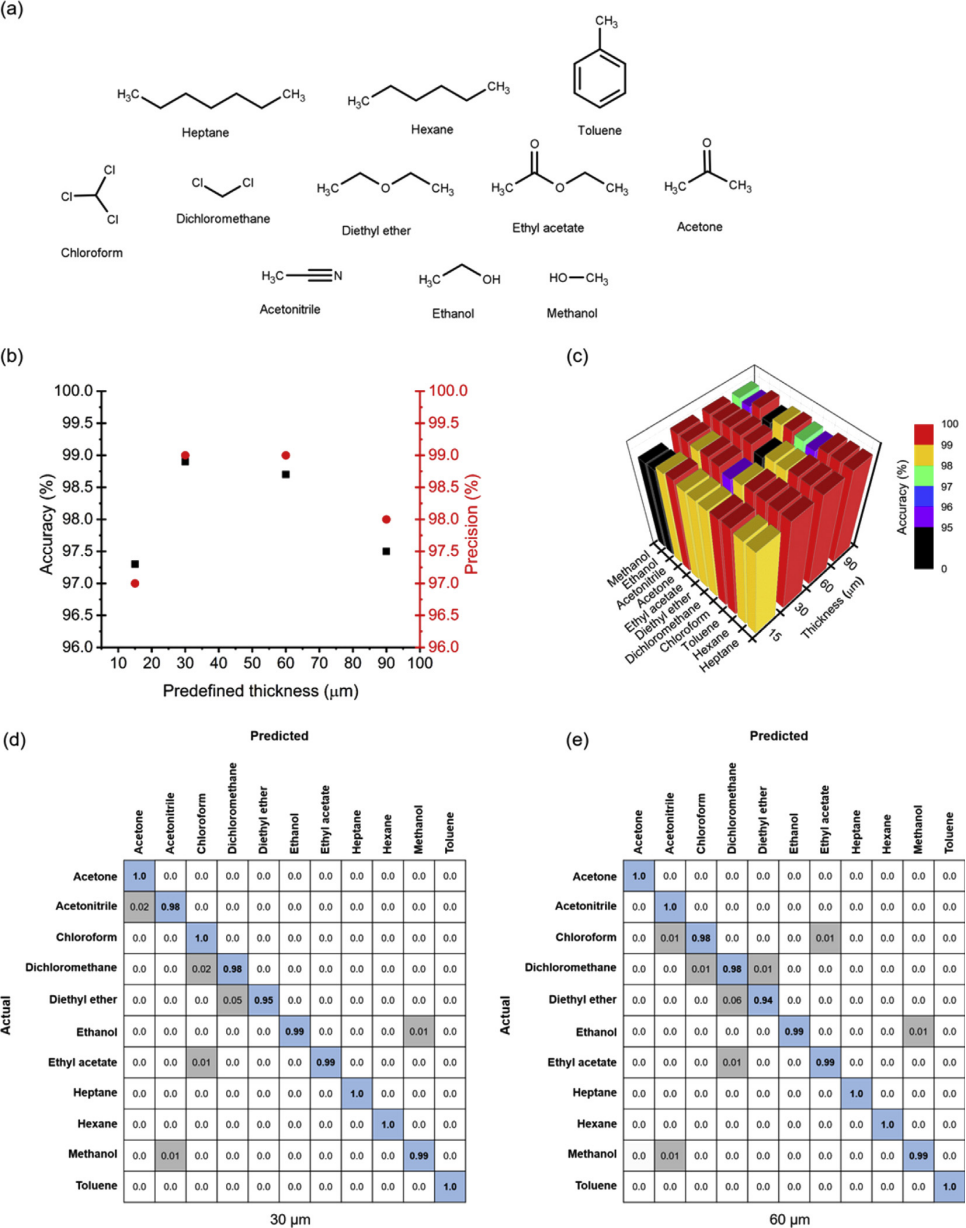
Accuracy and precision for the identification of 11 volatile organic compounds (VOCs) based on the signals obtained from the hybrid gel films with distinct thicknesses. a) Chemical structures of the 11 VOCs used in this study. b) Overall accuracy and precision. c) Correct prediction of the support vector machines (SVM) classifier for the hybrid gel films with different thicknesses. d) Confusion matrix for SVM, illustrating the prediction results regarding 11 VOCs for the 30-μm-thick and e) 60-μm-thick hybrid gel films. Blue squares in the diagonal represent the correct predictions made by the classifier, and gray squares represent the incorrect predictions.

The effect of film thickness on the gas-sensing performance of hybrid gels was further evaluated in an in-house tailor-made e-nose (Fig. 1d), through the sequential exposure of films with 15 μm, 30 μm, 60 μm, and 90 μm to 11 VOCs from distinct chemical classes (Fig. 5a). Sensor responses were fast and reversible, and each VOC yielded typical signals, as observed in Fig. 4. After signal collection, powerful machine learning tools were used to analyze the data. Signal features, namely morphological features and parameters of curve fitting models previously selected through computational methods (Fig. S4), were used as input variables for an SVM-based classification algorithm. SVM is a supervised machine learning method as the algorithm learns patterns in the inputs based on training (the presentation of inputs and known outputs). In other words, the algorithm learns the model that maps inputs into output and will be able to make further predictions on unknown inputs. When the outputs are classes, it is a classification problem (the one we present in this work). Confusion matrices summarize the automatic classification results of SVM applied to unknown inputs. The lines in confusion matrices represent the true VOC, known *a priori*, and the columns correspond to the predicted VOC. The diagonal corresponds to the percentage of correct VOC predictions and represents the accuracy of the classifier toward the VOC associated with each diagonal cell.

Accuracy and precision scores obtained for the different thicknesses in the study are shown in Fig. 5 and Fig. S10. The highest overall accuracy score (98.9%) was registered for the gelatin hybrid film thickness of 30 μm, with a precision of 99.0%, followed by 60-μm-thick films with 98.8% accuracy, and finally, 15-μm-thick and 90-μm-thick films with accuracy scores of 97.3% and 97.5%, respectively. When looking at the accuracy of the prediction for each individual VOC, 30-μm-thick and 60-μm-thick films perfectly classify acetone, hexane, heptane, and toluene out of the 11 VOCs tested, also visible in the respective confusion matrices (Fig. 5c). Interestingly, toluene is the only VOC classified with 100% accuracy for all film thicknesses.

The 30 μm thickness appears as the best option for the production and use of gelatin-based gas-sensitive hybrid gels because of not only the more accurate predictions but also the reproducibility in film deposition and the lower amount of material needed when compared with the 60 μm thickness. This work shows, for the first time, that hybrid gels with a single composition (gelatin, [BMIM][DCA], 5CB, and water) and predefined thickness can predict the identity of 11 distinct VOCs with high accuracy when implementing a dedicated automatic classifier based on SVM for data processing.

## Conclusions

Currently, gelatin applications go far beyond the traditional fields of food, pharmaceutics, cosmetics, and photography. The remarkable structural and functional properties of gelatin and derived materials, together with their biocompatibility and biodegradability, easy chemical functionalization, and processing, give gelatin a noble position in the field of advanced functional materials. Gelatin is nowadays regarded as a material of excellence for three-dimensional and four-dimensional bioprinting of food and biomedical materials [10], [19], [20], [21], for developing functional textiles [22] and edible soft electronic components, including supercapacitors and (micro)electrodes for sensing [23], [24], for designing soft robots [11], as well as for acting as an interface platform between humans and electronic devices [25]. In this work, a novel application for gelatin is assessed through the use of hybrid gels, with interesting optical and electrical properties, in gas-sensing applications. Hybrid gels incorporate LCs, which are known to act as signal amplification materials for sensor development [26], [27], [28], [29], [30].

A single hybrid gel composition – gelatin, IL [BMIM][DCA], and LC 5CB – was studied in this work. The resulting hybrid gels were deposited in glass substrates as films with predefined thicknesses. The gels showed clear compartmentalization given by the encapsulation of LC molecules in droplets with predominant radial configuration. The optical properties of the material, arising from the anisotropic behavior of the LC droplets, were stable over time. The thickness of the film clearly influenced the optical properties of the hybrid gels and, consequently, their utility as optical gas-sensing materials. A direct correlation between film thickness and optical active area was observed, probably due to the higher number and diameter of the droplets in thicker films. The radial configuration of LCs inside the droplets could be easily disrupted in the presence of different VOCs, yielding typical VOC signatures. In this work, the selection of signal features to build the supervised learning algorithm was more sophisticated than the simple analysis of signal amplitude followed by PCA clustering [8], [9], [12]. In total, 12 different features were extracted from the signals and used as input data for the SVM classifier method. With this approach, highly accurate and precise VOC predictions (accuracy higher than 97%) were observed for all film thicknesses, in particular for the 30-μm-thick films. Using a gelatin film with a single composition (gelatin, [BMIM][DCA], 5CB, and water) instead of an array of materials with distinct compositions, it was possible to classify 11 VOCs from several chemical classes. Future work will comprise the application of the knowledge and tools developed in this work to evaluate the use of gelatin hybrid gels to identify volatile components within complex samples containing distinct VOC patterns. In addition, the limits of detection for VOCs or mixtures of VOCs will be measured in the context of particular target applications.

This work further strengthens the interest in stimuli-responsive biomaterials based on gelatin for developing sustainable electrooptical devices and in the application of machine learning methods in artificial olfaction.

## Conflicts of interest

The authors declare that they have no known competing financial interests or personal relationships that could have appeared to influence the work reported in this article.
