# Supplementary material for: Effect of film thickness in gelatin hybrid gels for artificial olfaction
Source: Mater Today Bio. 2019 Mar 22;1:100002. doi: 10.1016/j.mtbio.2019.100002 (PMC7061580; doi:10.1016/j.mtbio.2019.100002)
Supplement: Multimedia component 1 [file mmc1.docx]

Supplementary information

**Effect of film thickness in gelatin hybrid gels for artificial olfaction**

Carina Esteves^1&^, Gonçalo M. C. Santos^1&^, Cláudia Alves^1,2^, Susana I.C.J. Palma^1^, Ana R. Porteira^1^, João Filho^1^, Henrique M. A. Costa^1^, Vitor D. Alves^3^, Bruno M. Morais Faustino^4^, Isabel Ferreira^4^, Hugo Gamboa^2^, Ana C. A. Roque^1^*

^1^ UCIBIO, Departamento de Química, Faculdade de Ciências e Tecnologia, Universidade Nova de Lisboa, Caparica, Portugal

^2^ LIBPhys-UNL, Departamento de Física, Faculdade de Ciências e Tecnologia, Universidade Nova de Lisboa, Caparica, Portugal

^3^ LEAF – Linking Landscape, Environment, Agriculture and Food, Instituto Superior de Agronomia, Universidade de Lisboa, Lisboa, Portugal

^4^ CENIMAT/I3N, Departamento de Ciências dos Materiais, Faculdade de Ciências e Tecnologia, Universidade Nova de Lisboa, Caparica, Portugal

* Corresponding author

E-mail: [cecilia.roque@fct.unl.pt](mailto:cecilia.roque@fct.unl.pt) (ACAR)

^&^ These authors contributed equally to this work

**Supplementary Methods**

**Rheological properties -** For the characterization of the films´ rheological properties, the hybrid gel samples were prepared in an in-house 3D printed o-ring with 20mm inner diameter and 1mm height. Rheological measurements were carried out using a controlled stress HAAKE MARSIII rheometer (Thermo Scientific) with temperature controlled at 20ºC and using plate-plate serrated geometry with 20mm diameter. The viscoelastic properties were evaluated by carrying out stress sweeps within a stress range from 0.001 to 1000 Pa; frequency sweeps were performed at a constant tension within the linear viscoelastic region (20 Pa), in the frequency range 0.1-100Hz. During all the measures the gap was of 0.35mm.

**Scanning Electron Microscopy** - Morphological characterizations by scanning electron microscopy (SEM) were conducted on a Carl Zeiss AURIGA CrossBeam workstation coupled with energy dispersive X-ray spectroscopy. Hybrid gel films, prepared using an automatic film applicator for a predefined thickness of 90µm, were dried in the desiccator for two days. To avoid charge effects, thin films were previously coated with a 40nm Au/Pd conductive layer.

**Determination of amino residues** - The free amino groups of non-crosslinked and crosslinked gelatin matrices were determined through fluorescence titration experiments according with the protocol preciously described [3]. Briefly, in dark conditions, fluorescein isothiocyanate isomer I solution was prepared by dissolution in dimethyl sulfoxide and sodium bicarbonate buffer (50mM, pH≈8.5). Non-crosslinked and crosslinked multicomponent hybrid gel thin films were incubated in fluorescein freshly prepared solution for 40 minutes at room temperature. The thin films were then rinsed with miliQ,H_2_O and incubated in miliQ,H_2_O during 2 hours at 4ºC to remove unspecific bonded fluorescein molecules. Fluorescence of dried thin films was determined through fluorescence microscopy using a Microscope Zeiss Axio Observer.Z1/7 equipped with a filter 38 HE Green Fluorescent (Ex/Em: 450nm-490nm/500nm-550nm).

**Study of the films storage stability by POM** - To assess the morphological changes of the liquid crystal droplets in the gels upon storage at room conditions, defined regions of interest (ROI) of the hybrid gel films were followed during 6 months by POM. Crossed polarizers POM images were taken at 1, 2, 3, 4, 5, 16 and 24 weeks after gel’s production and compared regarding optical active area and morphological changes of the liquid crystal droplets. The 7 POM images of each ROI were first aligned using the TrackEM2 plugin [4] within FIJI (parameters: least squares mode and similarity transformation) and then cut to select only the common area of the aligned POM images. The aligned and cut POM images were then processed as follows. Optical active area variation in time was analyzed by comparing the mean grey value of the 7 POM images per ROI. To analyze morphological variations in time, the 7 POM images per ROI were first binarized (using FIJI) to highlight the areas occupied by liquid crystal droplets. Then, the relative variation of droplets position and size with time was identified through the “delta images”, which result from the pixel by pixel subtraction between each time point binary image and the binary image of week 1. To quantify such variation, the norm of the resulting “delta images” was calculated, according to equation 1:


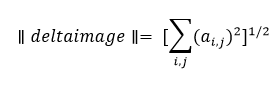
 (eq. S1)

$${\parallel deltaimage\parallel= [\sum_{i,j} {(a_{i,j})}^{2}]}^{1/2}$$

Where a_ij_ represents the pixel at line i and column j.

**Determination of liquid crystal droplets diameter** – The distribution of diameters of liquid crystal droplets in the films was determined using the panoramic POM images of the films optical active area (Fig. 1a). The diameter of a liquid crystal droplet was defined by its Feret’s diameter, the longest distance between any two points along a specified direction. Briefly, a crossed polarizers panoramic POM image of the optical active area was generated using the “Tiles” module of the ZEN Software. Afterwards, the panoramic image was processed using the macro “Find circles” and the Feret’s diameter of each object was determined using the “Analyze particles” function, both tools of FIJI distribution of ImageJ software[1][2]. A size filter was applied to the measurements to eliminate objects with diameter values less than 3.16µm (considered as noise - small defects of the gel that do not contribute to the optical response). The distribution of the liquid crystal droplet’s diameter within the film’s active optical area for 15µm, 30µm, 60µm and 90µm film thick was then represented in a histogram (Fig. S**2**).

**Estimation of the concentration of VOC sampled to the sensor’s chamber.** - VOC concentrations were indirectly measured through solvent evaporation. To estimate the amount of solvent loss by evaporation throughout time, the total solvent volume was divided in several reference points, measured using a ruler. The initial height of the solvent in the vial was considered as the first reference point; the subsequent points were equidistantly measured until the experiment reached ended (15 minutes). For characterizing each reference point, the solvent’s height in the vial, the solvent’s temperature, and the elapsed time since the previous measurement were registered.

The rationale of the method was based in the principle that, for a given decrease in solvent’s height in the vial, a correspondent volume was evaporated (Fig. S3).

For characterizing the system in each reference point, the liquid and vapor phases were both considered. It was assumed a binary water-VOC system, defined by the solvents’ purity – thus regarding the solvent as a solution – and that the totality of the evaporated solvent was expelled to the sensors chamber during the exposure periods, since the carrier gas flowrate (50 mL/s) is enough to push the total headspace volume of the solution’s chamber (≤ 27 mL). For characterizing the equilibrium in the liquid phase, the total mass (Eq. S2) and molar quantity of solvent and water (Eq. S3 – S4) were calculated:

$${mass}_{VOC}\left( g \right)=Volume \left( mL \right)\times Purity\% \left( \frac{v}{v} \right)\times\rho\left( \frac{g}{mL} \right) \left( eq. S2 \right)$$

$$n_{VOC}\left( mol \right)=\frac{{mass}_{VOC}\left( g \right)}{{Molar Mass}_{VOC} \left( \frac{g}{mol} \right)} \left( eq. S3 \right)$$

$$n_{H_{2}O}\left( mol \right)=Volume \left( mL \right)\times\frac{1-Purity \% \left( \frac{v}{v} \right)}{{Molar Mass}_{H_{2}O}\left( \frac{g}{mol} \right)} \left( eq. S4 \right)$$

where ρ corresponds to the solvent’s volumetric mass density under NPT conditions.

Then, the solvent and water liquid molar fractions, $x_{VOC}$ and $x_{H_{2}O}$, were obtained (Eq. S5):

$$Molar Fraction=\frac{{Moles}_{i}}{Total moles}, i=organic solvent, water \left( eq. S5 \right)$$

By estimating the volume of solvent evaporated between reference points (Eq. S6), the total mass and molar quantity of evaporated solvent and water were similarly calculated through Equations S2 – S4, but accounting for the change in volume. By dividing the volume of evaporated solvent by the total duration of the exposure periods between reference points (Eq. S7), an approximate volatilization rate was obtained (Eq. S8). A similar calculation can be made for the molar and mass quantities. It was thus assumed that the volatilization rate remained constant during the total exposure period.

$${Volume}_{Evaporated}(mL)=\pi\times(r-2e)^{2}\times(\Delta h-e) (eq. S6)$$

where *r* represents the vial radius, *e* the vial’s thickness and *Δh* the difference in liquid height between reference points.

$$Total Exposure Time \left( s \right)=\frac{\Delta t \left( s \right)}{30 \left( \frac{s}{cycle} \right)}\times5\left( \frac{s}{exposure period} \right) \left( eq. S7 \right)$$

where *Δt* represents the time elapsed between reference points, thus giving the total number of cycles when divided by the its total duration – considering one cycle equal to one exposure plus one recovery period.

$$Volatilization Rate \left( \frac{mL}{s} \right)=\frac{{Volume}_{Evaporated}\left( mL \right)}{Total Exposure Time \left( s \right)} \left( eq. S8 \right)$$

For characterizing the gas phase, the vapour pressure of the solvents was calculated using the Antoine Equation (Eq. S9):

$${Antoine Equation: P}_{Solvent}^{*}={10}^{A-\frac{B}{C+T}}, P\left( bar \right), T\left( ºC \right) \left( eq. S9 \right)$$

where the coefficients A, B and C were obtained through tabulated values[5] (Table S1), specific for each solvent at about 298 Kelvin. *T* represents the solvent’s temperature.

Table S1- VOCs' Antoine's constants and heat capacities.

|  | Solvents | | | | | | |
| --- | --- | --- | --- | --- | --- | --- | --- |
| Antoine's constants | (C_2_H_5_)_2_O | C_6_H_14_ | C_2_H_5_OH | CH_2_Cl2 | C_4_H_8_O_2_ | C_3_H_6_O | C_7_H_8_ |
| A | 6.96559 | 4.00091 | 4.92365 | 4.07622 | 4.21248 | 4.35647 | 4.0854 |
| B | 1071.54 | 1171.17 | 1410.46 | 1070.07 | 1238.15 | 1277.03 | 1348.77 |
| C | 227.774 | 224.408 | 208.514 | 223.24 | 217.205 | 237.23 | 219.976 |
| Heat capaciity (cal/g.K) | 0.556 | 0.735 | 1.375 | 0.746 | 0.719 | 1.091 | 0.687 |

The vapor pressure of water was taken from tabulated values[5] at room temperature and the water bath’s temperature (Table S2). The solutions’ water and solvent partial pressures were calculated using Raoult’s Law (Eq. S10):

$$Raoult^{'}s Law: P_{i}=x_{i}\times P_{i}^{*} \left( bar \right), i=Solvent, water \left( eq. S10 \right)$$

Since the mixture of gas from the solution headspace and from the ambient air (carrier gas) may be at different temperatures, the temperature of the resulting mixture (Eq. S11) was determined to allow further calculations:

$$\left\{ \begin{aligned} \boldsymbol{Q}_{\boldsymbol{Carrier}} \left( \frac{\boldsymbol{cal}}{\boldsymbol{s}} \right)={mass}_{carrier}\left( \frac{g}{s} \right)\times{cp}_{carrier}\left( \frac{cal}{g.K} \right)\times\left( \boldsymbol{T}_{\boldsymbol{Mixture}}-T_{Carrier initial} \right)\left( K \right) \\ \boldsymbol{Q}_{\boldsymbol{Solution}} \left( \frac{\boldsymbol{cal}}{\boldsymbol{s}} \right)={mass}_{solution}\left( \frac{g}{s} \right)\times{cp}_{solution}\times\left( \boldsymbol{T}_{\boldsymbol{Mixture}}-T_{Headspace initial} \right)\left( K \right) \end{aligned} \right. \left( eq. S11 \right)$$

where ${mass}_{carrier}$ represents the flow rate of carrier gas, ${mass}_{solution}$ the average value of the solution’s volatilization rate measured at each reference point, Q the sensible heat and cp the heat capacity of the solvent or of the external air (Tables S1 – S2), obtained through tabulated values. Establishing${|Q}_{Carrier}\left| ={|Q}_{Solution} \right|$, the temperature of the mixture was calculated. It was observed that the temperature of the mixture always approached that of the external conditions.

For converting the liquid phase rate of volatilized volume onto the gas phase, equation S12 was employed

$${Volume}_{gas phase volatilization rate}\left( \frac{L}{s} \right)=\frac{n_{VOC}\left( \frac{mol}{s} \right)\times R\left( L.\frac{bar}{mol.K} \right)\times T_{Mixture} \left( K \right)}{P_{VOC}\left( bar \right)} \left( eq. S12 \right)$$

where *n_VOC_* is the rate of moles of evaporated solvent from the liquid phase and *P_VOC_* the solvent’s partial pressure.

Finally, for estimating the concentrations of VOCs sampled to the e-nose (Table S3), the volatilization rate (Eq. S12) was divided by the total gas mixture in the carrier gas flow rate (Eq. S13).

$${Concentration}_{VOC} \% \left( \frac{v}{v} \right)=\frac{V_{VOC}\left( \frac{\mu L}{s} \right)}{V_{VOC}\left( \frac{L}{s} \right)+V_{Carrier Gas}\left( \frac{L}{s} \right)}\left( ppm \right)\times{10}^{-4}\left( \frac{\%\left( \frac{v}{v} \right)}{ppm} \right) \left( eq. S13 \right)$$

Table S2- Other constants used for thermodynamics characterization.

| Ideal Gas Constant, R (L.bar/mol.K) | 0.08206 |
| --- | --- |
| Heat Capacityair (cal/g.K) | 0.445 |
| Water vapour pressureRoom Conditions (bar, 20 ºC) | 0.02275 |
| Water vapour pressureSolution (bar, 24 ºC) | 0.02912 |

Table.S3- Concentrations of VOCs sampled to the e-nose sensors chamber.

| **VOC** | **Average sampled VOC concentration %(v/v)** |
| --- | --- |
| Toluene | 12 |
| Ethanol | 13 |
| Dichloromethane | 13 |
| n-Hexane | 14 |
| Ethyl Acetate | 14 |
| Acetone | 15 |
| Diethyl Ether | 15 |
| n-Heptane | 13 |
| Chloroform | 15 |
| Acetonitrile | 13 |
| Methanol | 14 |

**Supplementary Figures**


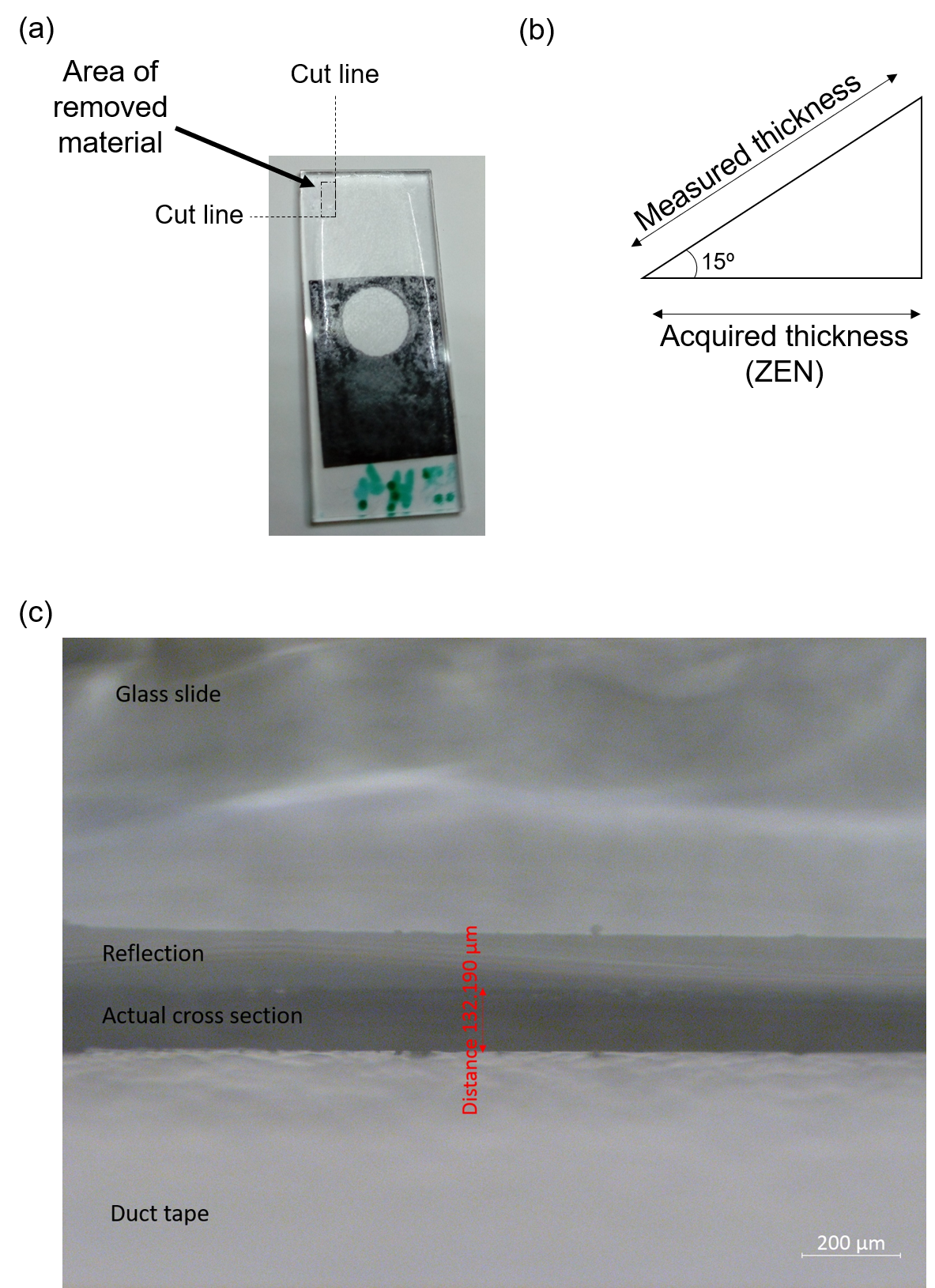


Fig. S1 – Image showing the method followed to measure the film´s thickness. (a) represents the area of the film removed, (b) shows the angle of 15º used for the determination of the “measured thickness” according to Equation 1 in main text. (c) shows the validation of the method using as control sample a cross section of an electrical isolator duct tape with a thickness of 130µm, as stated by the supplier (3M). In this case, the glass slide is clean, thus a reflection can be observed. Different grey tones help in identifying the borders of the duct tape cross-section.





Fig. S2 – Histogram of the distribution of liquid crystal droplet’s diameter within the film’s active optical area for 15µm, 30µm, 60µm and 90µm film thickness.


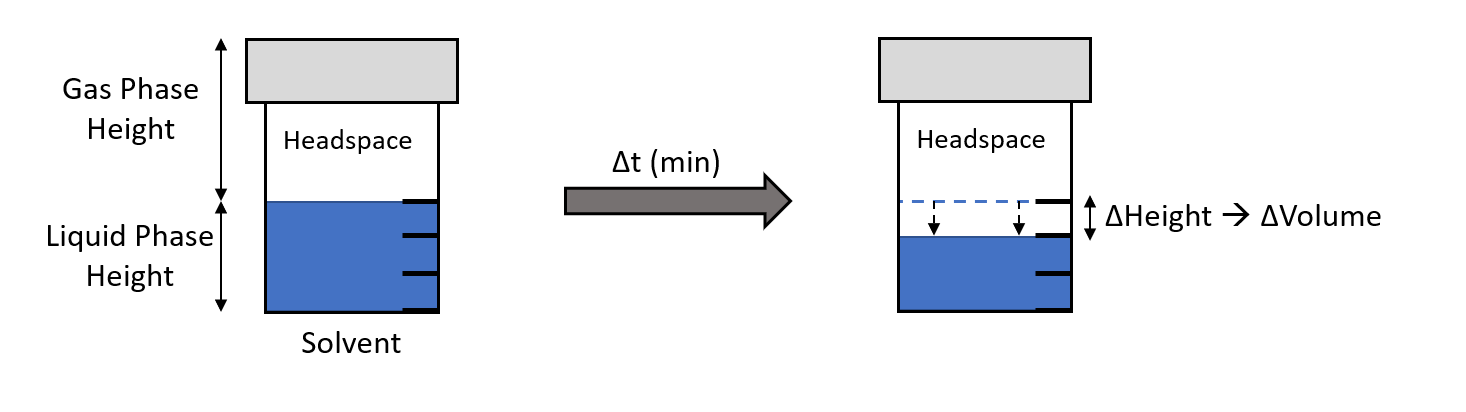


Fig. S3 – Schematic representation of the measurement of solvent’s volume loss by evaporation throughout time.

**
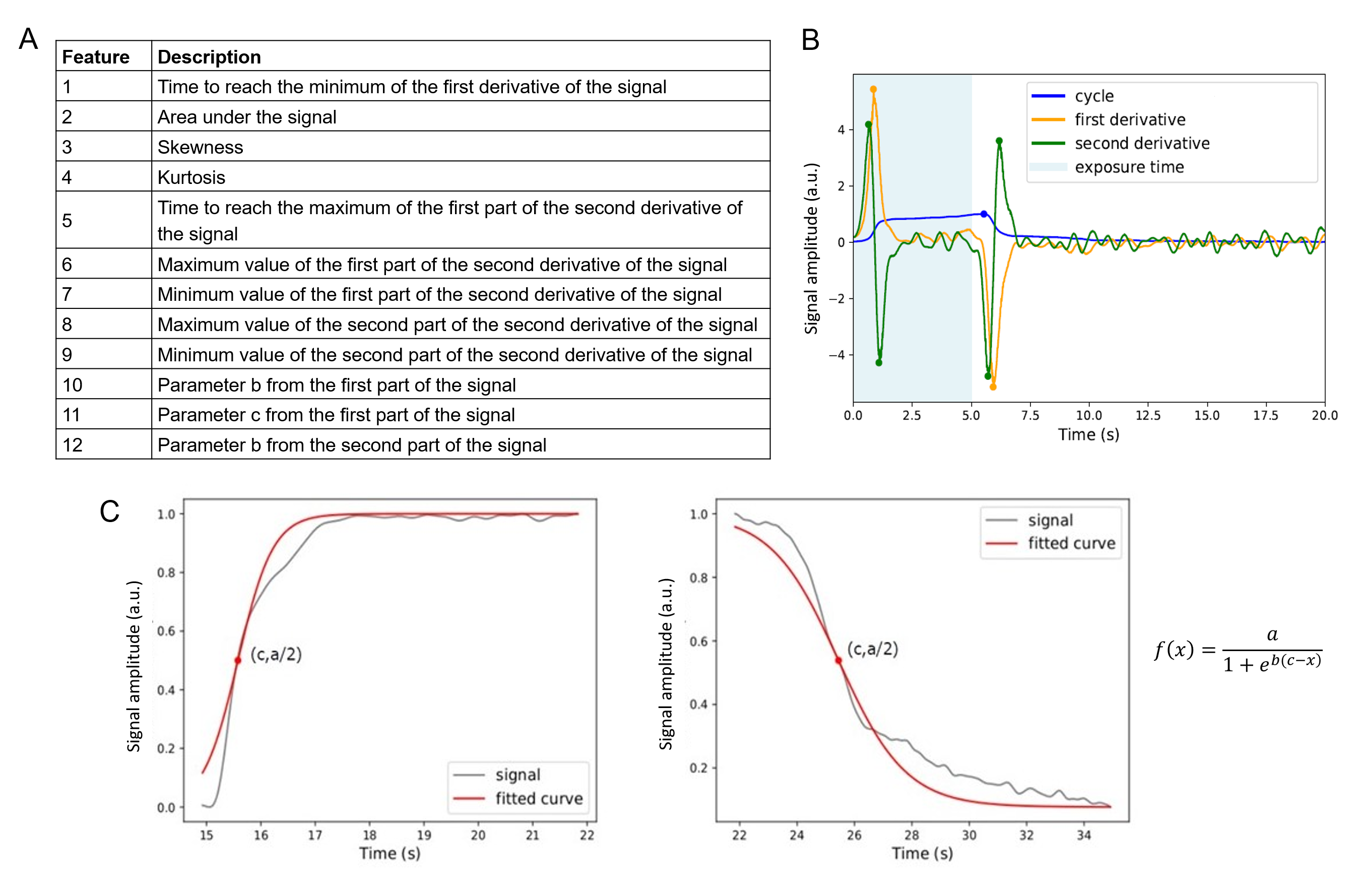
**

Fig. S4 - Schematic representation of the optical signal produced by the gel thin films and features used as input for the SVM classifier. (A) Description of the 12 features extracted from the signals and used as input for the implemented SVM classifier. (B) Typical waveform of an exposure/recovery cycle from the optical signal and its derivatives, highlighting the respective maxima and minima. (C) Illustration of the procedure for fitting a cycle to the logistic function, where the signal is divided in two parts, each of which is fitted individually to the function defined by the represented equation; parameters a and c are derived from the signal, while parameter b is obtained from the fitting.


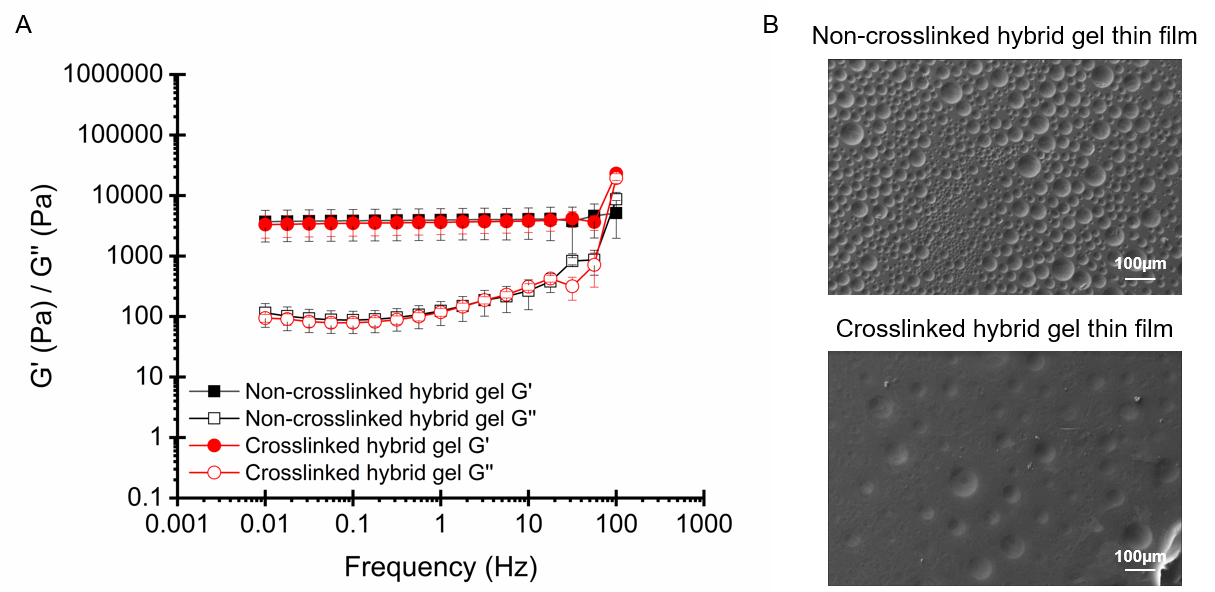


Fig. S5 - (A) Mechanical properties of non-crosslinked and crosslinked hybrid gels: surface shear storage (G’) and loss (G’’) moduli as a function of frequency (n=2). (B) Scanning electron microscope images: surface of the non-crosslinked and crosslinked hybrid gels.

**
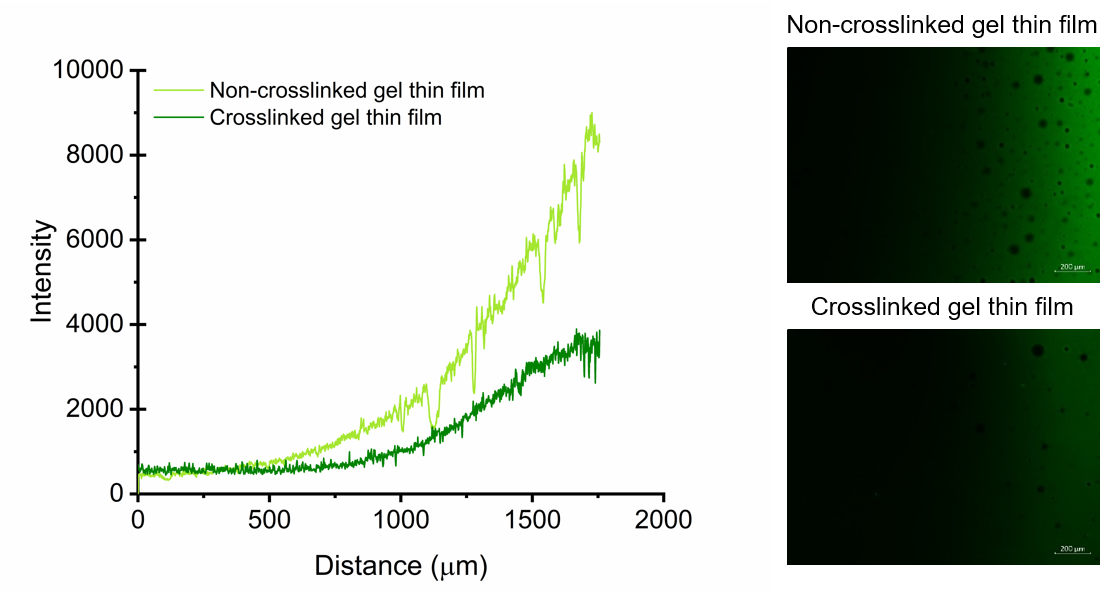
**

Fig. S6 - Fluorescence intensity and optical fluorescence images of non-crosslinked and crosslinked hybrid gel films. Results show the effective crosslinking of gelatin by the reduction of FITC labeling, which corresponds to a reduction in free amines.


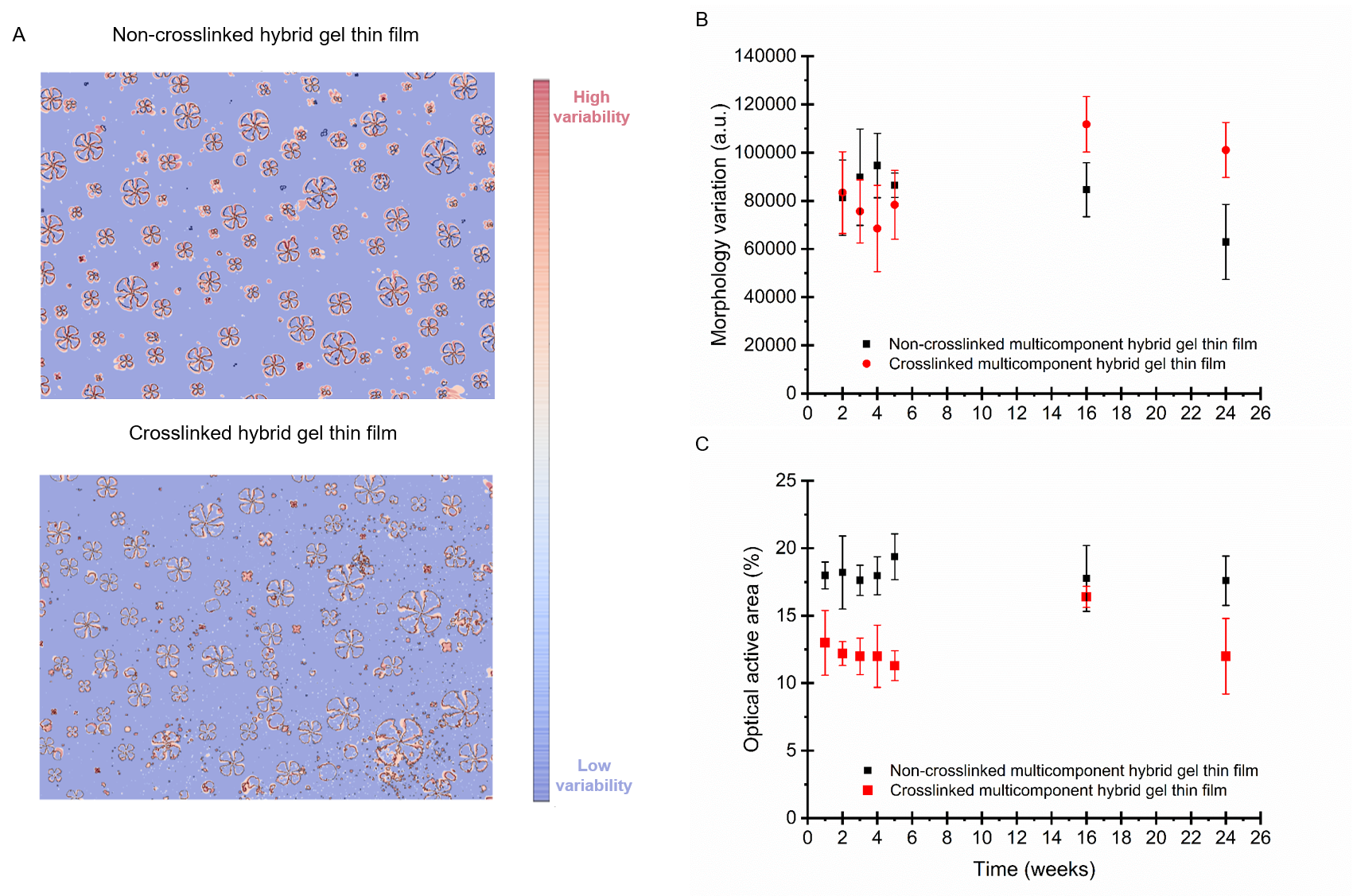


Fig. S7 – Morphology variation of non-crosslinked and crosslinked hybrid gel films along 24 weeks: (A) crossed polarized optical microscope images over position (B) morphology variation quantification and (C) variation of optical active area (n=3).


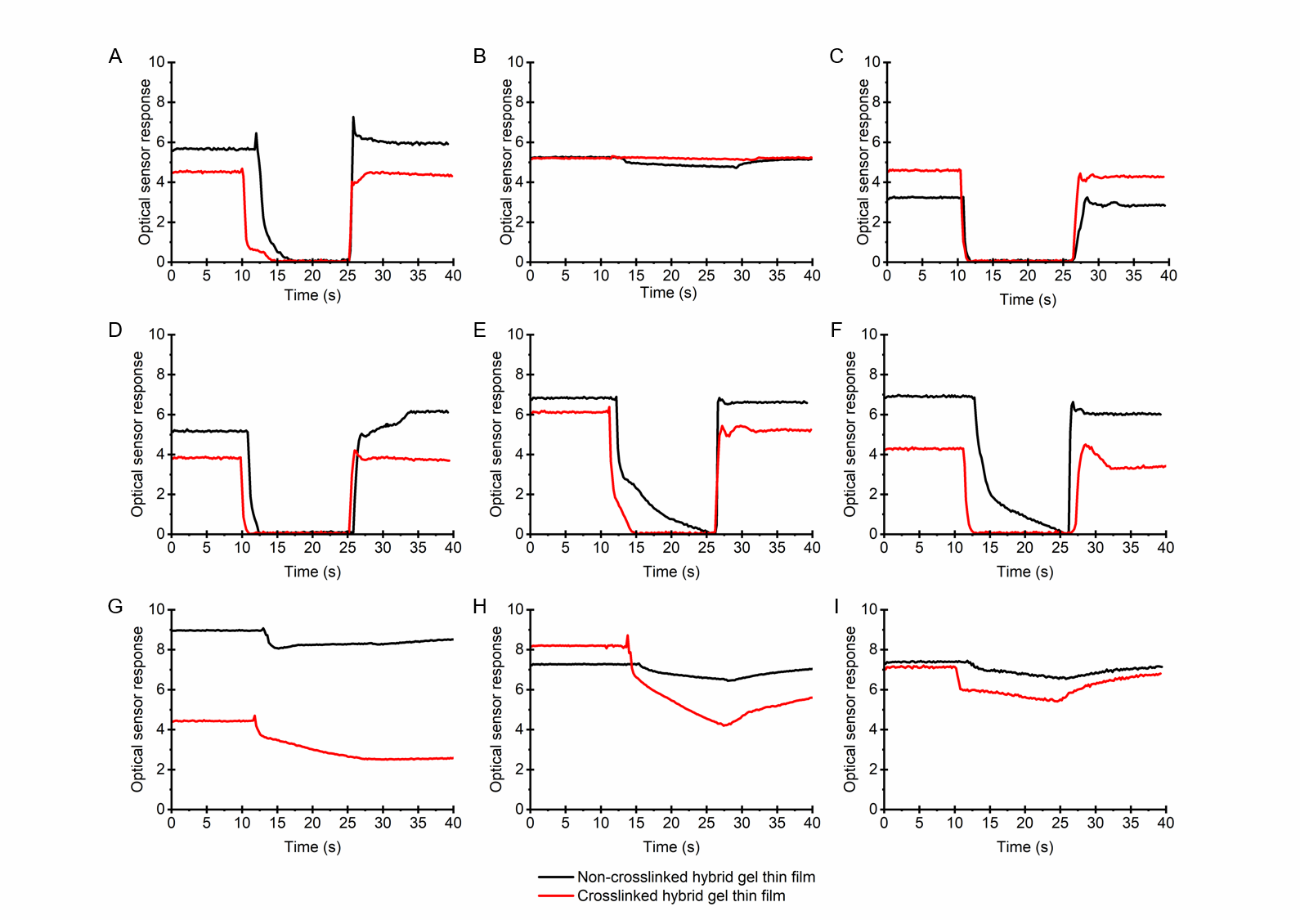


Fig. S8 - Optical sensor response, under crossed polarizers, of non-crosslinked and crosslinked hybrid gel films to nine volatile organic compounds: (A) hexane, (B) toluene, (C) dichloromethane, (D) diethyl ether, (E) ethyl acetate, (F) acetone, (G) acetic acid, (H) ethanol, (I) isopropanol. Volatile organic compound exposition at 10s; recovery pump at 25s.


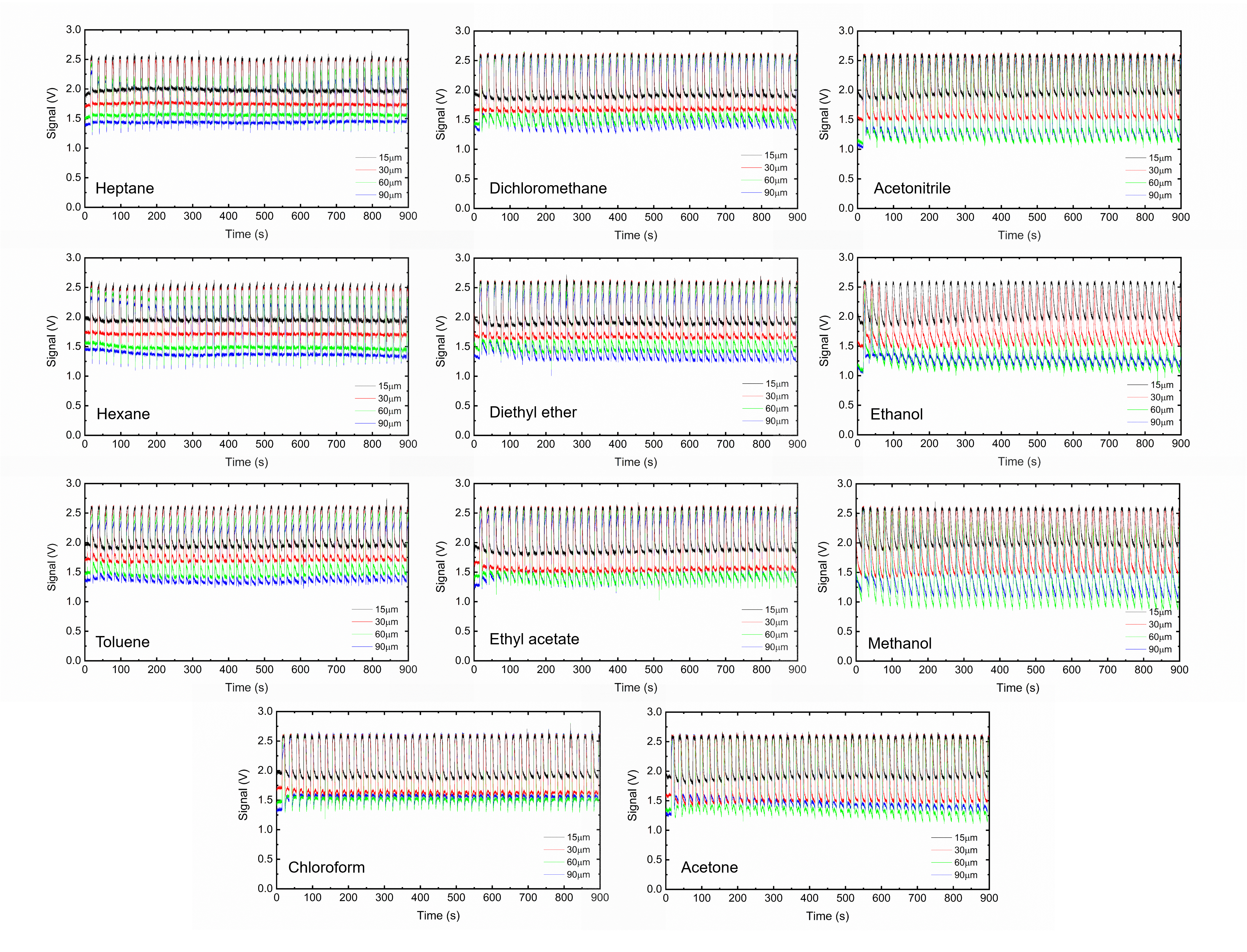


Fig S9 - Signals obtained by the e-nose custom built device upon exposure of gelatin hybrid gel films with defined thicknesses to 11 different volatile organic compounds.


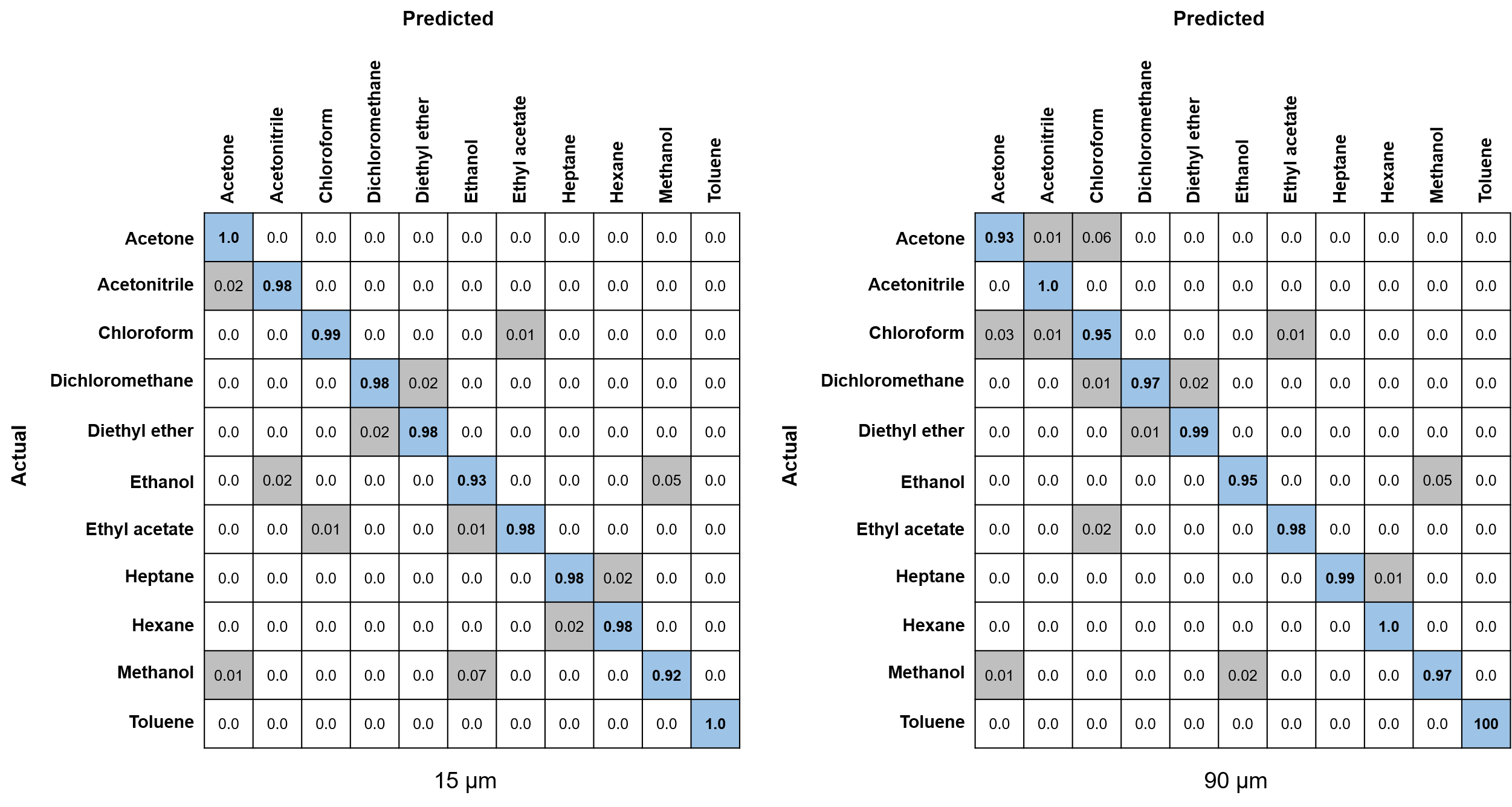


Fig S10 – Normalized confusion matrices illustrating the prediction results of the SVM classifier towards 11 different volatile organic compounds when the optical signals of the 15µm and 90µm films were used as inputs. Calculated average accuracy for 15 µm films was 99.7% while for 90 µm films was 99.8%. ◼ represent the correct predictions made by the classifier and ◼ represent the incorrect predictions.

Video S1 – Video recorded in the polarized optical microscope (polarizers crossed at 90º) after exposing a gelatin film control (composed of gelatin, liquid crystal and water) to hexane.

Video S2 - Video recorded in the polarized optical microscope (polarizers crossed at 90º) after exposing a gelatin hybrid gel film to hexane.

**References**

[1] J. Schindelin, I. Arganda-Carreras, E. Frise, V. Kaynig, M. Longair, T. Pietzsch, S. Preibisch, C. Rueden, S. Saalfeld, B. Schmid, J. Tinevez, D.J. White, V. Hartenstein, K. Eliceiri, P. Tomancak, A. Cardona, Fiji : an open-source platform for biological-image analysis, Nat. Methods. 9 (2012). doi:10.1038/nmeth.2019.

[2] C.T. Rueden, J. Schindelin, M.C. Hiner, B.E. Dezonia, A.E. Walter, E.T. Arena, K.W. Eliceiri, ImageJ2 : ImageJ for the next generation of scientific image data, BMC Bioinformatics. 18 (2017) 1–26. doi:10.1186/s12859-017-1934-z.

[3] S. Farris, J. Song, Q. Huang, Alternative reaction mechanism for the cross-linking of gelatin with glutaraldehyde, J. Agric. Food Chem. 58 (2010) 998–1003. doi:10.1021/jf9031603.

[4] A. Cardona, S. Saalfeld, J. Schindelin, I. Arganda-carreras, S. Preibisch, M. Longair, P. Tomancak, V. Hartenstein, R.J. Douglas, TrakEM2 Software for Neural Circuit Reconstruction, PLoS One. 7 (2012) e38011. doi:10.1371/journal.pone.0038011.

[5] P. S, R.H. Perry, D.W. Green, J.O. Maloney, Chemical Engineers ’ Handbook, Seventh, McGraw-Hill Companies, 1997. doi:10.1021/ed027p533.1.
